# Editorial: New insights into the role of tumor microbial microenvironment in tumor immunotherapy

**DOI:** 10.3389/fcimb.2024.1384645

**Published:** 2024-02-26

**Authors:** Peixin Dong, Xu Chen, Jianguo Tang, Wei Wang

**Affiliations:** ^1^ Department of Obstetrics and Gynecology, Hokkaido University School of Medicine, Hokkaido University, Sapporo, Japan; ^2^ Center of Clinical Laboratory, The First Affiliated Hospital of Soochow University, Suzhou, Jiangsu, China; ^3^ Shanghai Fifth People’s Hospital, Fudan University, Shanghai, China; ^4^ Department of Thoracic Surgery, The First Affiliated Hospital of Nanjing Medical University, Nanjing, China

**Keywords:** tumor microbial microenvironment, microbial metabolites, tumor viruses, microbiome, tumor immunotherapy

The intricate relationship between the tumor microenvironment (TME) and its microbial components is becoming acknowledged as a crucial factor influencing the success of cancer immunotherapy. The Research Topic titled “*New insights into the role of tumor microbial microenvironment in tumor immunotherapy*” features a collection of six insightful papers that delve into this complex interplay, providing novel revelations and advancing the field.

In a study by Liu et al., machine learning algorithms were deployed to discern key determinants of survival in gastric cancer patients undergoing radical surgery and immunotherapy. Key risk factors affecting survival at 1-, 3-, and 5-year intervals post-treatment included advanced age, tumor characteristics, and specific biomarkers such as CEA, CA125, CA72-4 levels, and H. pylori infection. This research underscores the prognostic significance of variables such as the presence of H. pylori infection. This investigation exemplifies the transformative potential of algorithmic analytics in refining patient-specific therapeutic strategies and enhancing the precision of clinical prognostication.

The critical analysis by Han et al. delves into the gut microbiome’s influential role in sculpting the colorectal cancer (CRC) microenvironment. The authors meticulously unravel how microbial entities and their metabolic outputs can modulate the transition from benign adenomatous polyps to invasive carcinomas, with implications for the genomic integrity of epithelial cells lining the intestine. This review accentuates the pivotal role of gut microbiota profiling in the anticipatory detection of CRC and the bolstering of patient survival, thereby establishing the gut microbiome as an axis of therapeutic innovation.

Huang et al. present an exhaustive review of the dialogue between intratumor bacteria and neoplastic cells. Their investigation illuminates the mechanisms by which bacterial colonies within tumors can modulate oncogenesis, metastatic potential, and the host’s response to anticancer therapies. Deciphering this intricate bacterial-host interplay is imperative for the conception of more efficacious therapeutic modalities.

Gong et al. examine the consequences of HPV-induced metabolic alterations in the TME of head and neck squamous cell carcinoma (HNSCC). Their analysis sheds light on how HPV disrupts immune monitoring and the progression of cancer cells, influencing the effectiveness of immune-based treatments. Their research highlights the potential of targeting the interplay between HPV oncogenic activity and the TME to enhance the impact of immunotherapy in HNSCC.

Cao et al. investigate the impact of pulmonary bacterial infections on the efficacy of immunotherapy in non-small cell lung cancer (NSCLC). Their thorough evaluation demonstrates that bacterial lung infections (BLI) may serve as a predictive factor for better outcomes in NSCLC patients undergoing immune checkpoint inhibitor therapy, although limitations such as its retrospective design and lack of data on BLI bacteria types warrant further research.

In their study on thyroid oncology, Zhu et al. utilized Mendelian randomization to investigate the causal link between gut microbiota and thyroid cancer (TC), identifying seven microbial taxa significantly associated with TC risk. The analysis revealed that certain genera, such as Butyrivibrio and Fusicatenibacter, increased TC risk, while others like Olsenella were protective against TC. The authors provide compelling evidence linking alterations in the gut microbiome to thyroid cancer pathogenesis, positing gut microbiome modulation as a viable avenue for cancer prevention and therapy.

Collectively, these contributions deepen our grasp of the tumor microbial microenvironment and its profound impact on cancer immunotherapy. They herald a trajectory for research that may culminate in more tailored and effective oncological treatments. As we continue to demystify the complexities of the TME and its microbial partners, we edge toward an innovative epoch of oncology that leverages the innate capabilities of the immune system, informed by the revelations of these trailblazing investigations.

## Author contributions

PD: Writing – original draft, Writing – review & editing. XC: Writing – review & editing. JT: Writing – review & editing. WW: Conceptualization, Data curation, Formal analysis, Funding acquisition, Investigation, Methodology, Project administration, Resources, Software, Supervision, Validation, Visualization, Writing – review & editing.

